# Quality and Accessibility of Liquid Biopsy Information

**DOI:** 10.1001/jamanetworkopen.2024.10171

**Published:** 2024-05-07

**Authors:** Henry K. Litt, Emma Greenstreet-Akman, Evelin Trejo, Narjust Florez, Ana I. Velazquez

**Affiliations:** 1 Department of Medicine, University of California, San Francisco, San Francisco; 2 School of Medicine, University of California, San Francisco, San Francisco; 3 Department of Medicine, Division of Hematology/Oncology, University of California, San Francisco, San Francisco; 4 Helen Diller Family Comprehensive Cancer Center, University of California, San Francisco, San Francisco; 5 Department of Medical Oncology, Dana-Farber Cancer Institute, Boston, Massachusetts; 6 Harvard Medical School, Boston, Massachusetts

## Abstract

This cross-sectional study evaluates the information on a circulating tumor DNA test available to the public on popular internet resources.

## Introduction

Circulating tumor DNA assays, known as liquid biopsies, are increasingly used to genotype cancers, guide therapy selection, and identify resistance mechanisms to cancer therapy.^1^ As more patients undergo liquid biopsies, it is essential that they can understand the benefits and limitations. Patients often obtain medical information on the internet or social media. Because online medical information can be inaccurate, biased, and difficult to comprehend,^2^ we evaluated the quality, accessibility, and understandability of online information about liquid biopsies.

## Methods

We performed an online search of *liquid biopsy* using a search engine (Google; Alphabet) and social network (YouTube; Alphabet) and conducted a cross-sectional analysis of the top videos and web page results. Videos over 5 minutes, scientific articles, and non–English language content were excluded. The University of California, San Francisco Institutional Review Board approved this cross-sectional study and waived the informed consent requirement as the work was not human participant research. We followed the STROBE reporting guideline.

Two independent reviewers (H.K.L., E.G.A.) evaluated each video for characteristics, perceived speaker identities and roles, and health information presented. Understandability and quality of consumer health information were evaluated using validated tools (PEMAT [Patient Education Materials Assessment Tool], DISCERN).^3,4^ Interobserver agreement between reviewers was 87%. Disagreements (n = 26) were resolved by another independent reviewer (E.T.).

Web pages were evaluated for accessibility (visual aids, languages available, reading level), quality of information (authors, citations, publication year, indications, limitations), discussion of cost or insurance coverage, and presence of commercial bias. Reading level was evaluated using validated indices: Flesch-Kincaid Grade Level, Gunning Fog, and Simple Measure of Gobbledygook (SMOG) Index.^5^ Commercial bias included disclosure of conflicts of interest, primary company websites, and presence of branding or funding from for-profit corporations.

Descriptive statistics were calculated using Stata, version 17 (StataCorp LLC). Analyses were performed between November 2022 and February 2023.

## Results

We analyzed 100 liquid biopsy videos, with median (IQR) views per video of 190 (75-719). Video speakers lacked diversity, with 82 (65.6%) perceived as male and 39 (31.2%) as female and 98 (78.4%) as non-Hispanic White (Table 1). These videos were complex and of low quality. Only 9.0% of videos were understandable (PEMAT score: ≥70%), and 8.0% presented moderate to high quality health information (DISCERN score: 3-5).

**Table 1.** Characteristics of Liquid Biopsy Videos and Featured Speakers

Characteristic	No. (%)
No. of videos	100
No. of featured speakers	125
Understandability or quality measures	
Understandable (PEMAT score: ≥70%)^a^	9 (9.0)
Moderate to high quality (DISCERN score: 3-5)^b^	8 (8.0)
Video creator	
Academic journal or society	38 (38.0)
Media organization	29 (29.0)
Health care industry company	12 (12.0)
Hospital system	11 (11.0)
Other^c^	10 (10.0)
Intended audience	
Health care practitioner or scientific community	72 (72.0)
General public	28 (28.0)
Type of speaker or narrator	
Physician	77 (61.6)
Basic science researcher	26 (20.8)
Industry	9 (7.2)
Media group	6 (4.8)
Patient	4 (3.2)
Other or unknown^d^	3 (2.4)
Perceived race and ethnicity of speaker	
Asian	22 (17.6)
Black	1 (0.8)
Hispanic or Latino	1 (0.8)
Non-Hispanic White	98 (78.4)
Unknown	3 (2.4)
Perceived sex of speaker	
Male	82 (65.6)
Female	39 (31.2)
Unknown	4 (3.2)

Abbreviation: PEMAT, Patient Education Materials Assessment Tool.

^a^ PEMAT score range: 0%-100%, with the highest score indicating perfect understandability.

^b^ DISCERN score range: 0-5, with the highest score indicating minimal shortcomings as a source of patient information.

^c^ Other video creator included research organization and general population.

^d^ Other type of speaker or narrator included general population.

We reviewed 100 web pages about liquid biopsies, of which none had reading levels of sixth grade or lower, as recommended by medical society guidelines^2^ (Table 2). Median (IQR) Flesch-Kincaid Grade Level, Gunning Fog Index, and SMOG Index readability scores were 14.1 (12.1-15.8), 16.4 (14.4-18.6), and 15.9 (13.9-16.9), respectively, which are equivalent to college-level education. Web pages rarely promoted accessibility through visual aids (20.0%) or multiple languages (5.0%). Sixty-one percent of web pages had commercial biases, and less than half included author names (42%), citations (29%), and mentions of indications (36%) or limitations (35%) of liquid biopsies.

**Table 2.** Characteristics of Liquid Biopsy Web Pages

Characteristic	No. (%)
No. of web pages	100
Web page creator	
Health care industry company	35 (35.0)
Academic society or journal	19 (19.0)
Hospital system	18 (18.0)
Media organization	18 (18.0)
Other	10 (10.0)
Accessibility	
Visual aid present	20 (20.0)
Multiple languages available	5 (5.0)
Quality	
Authors’ names present	42 (42.0)
Citation present	29 (29.0)
Publication year present	57 (57.0)
Indications mentioned	36 (36.0)
Limitations mentioned	35 (35.0)
Cost or insurance coverage mentioned	16 (16.0)
Commercial bias	61 (61.0)
Corporation brand mentioned	50 (50.0)
Liquid biopsy company website	29 (29.0)
Company logo present	27 (27.0)
Conflict of interest mentioned	5 (5.0)
Funded by liquid biopsy company	3 (3.0)
Reading level, median (IQR) text readability	
Flesch-Kincaid Grade Level	14.1 (12.1-15.8)
Gunning Fog Index	16.4 (14.4-18.6)
SMOG Index	15.9 (13.9-16.9)

Abbreviation: SMOG, Simple Measure of Gobbledygook.

## Discussion

We found that liquid biopsy videos and web pages had extensive shortcomings as sources of patient health information. Consistent with previous research assessing cancer-related online materials,^2^ most patients need help understanding online content about liquid biopsies and may be more confused after searching online. Health literacy plays an important role in patient experiences and outcomes. Patients with cancer often report knowledge gaps about their care and may be unable to clarify their concerns with clinicians, necessitating accessible resources to make informed decisions.^6^ It is crucial that patients understand emerging technologies, such as liquid biopsies, as their potential benefits, costs, and unintended adverse effects are being discovered.^1^

Study limitations include lack of longitudinal evaluation of available information, exclusion of lengthy and non–English language content, lack of blinding of reviewers to the study’s purpose, and potential reviewers’ biased perceptions of speaker demographics (vs self-identification). With increasing propagation of medical misinformation online, efforts must be made to ensure that unbiased, high-quality information about novel diagnostic technologies and treatments is understandable and easily accessible to patients and caregivers.

## Notes

Supplement.
Data Sharing Statement
