# Supplementary material for: Quality and Accessibility of Liquid Biopsy Information
Source: JAMA Netw Open. 2024 May 7;7(5):e2410171. doi: 10.1001/jamanetworkopen.2024.10171 (PMC11077385; doi:10.1001/jamanetworkopen.2024.10171)
Supplement: Supplement. — Data Sharing Statement [file jamanetwopen-e2410171-s001.pdf]

Data Sharing Statement

Litt. Quality and Accessibility of Liquid Biopsy Information. *JAMA Netw Open*. Published May 07, 2024. doi:10.1001/jamanetworkopen.2024.10171

Data
Data available: Yes
Data types: Data (not involving human participants)
How to access data: Data requests should be directed to corresponding author.
When available: With publication

Supporting Documents
Document types: None

Additional Information
Who can access the data: anyone requesting the data
Types of analyses: for any purpose
Mechanisms of data availability: with investigator support
